# Carbonate Alkalinity Stress Induces Hepatopancreas Injury and Activates TLR2-MyD88-NF-κB-Related Responses in Chinese Mitten Crab

**DOI:** 10.3390/ani16131945

**Published:** 2026-06-23

**Authors:** Yichen Bai, Hongkun Guan, Yuhong Yang, Haoyang Sheng, Zhilin Jiang, Kangrun Liu, Changrui Fu, Peng Liu, Chenghui Yang

**Affiliations:** 1 College of Life Science and Technology, Harbin Normal University, Harbin 150025, China; baiyichen1621@163.com; 2 College of Animal Science and Technology, Northeast Agricultural University, Harbin 150030, China; 15546093978@163.com (H.G.); yuhongyang68@163.com (Y.Y.); 13039721273@163.com (H.S.); 18746012809@163.com (Z.J.); 13134282061@163.com (K.L.); 18246631326@163.com (C.F.); 3 Fisheries Research Institute, Harbin Academy of Agricultural Sciences, Harbin 150078, China

**Keywords:** Chinese mitten crab, carbonate alkalinity stress, hepatopancreas, apoptosis

## Abstract

Global freshwater salinization endangers aquatic species, yet its impacts on crustaceans remain poorly understood. This study investigated the hepatopancreatic response of *Eriocheir sinensis* to carbonate alkalinity stress (0, 4.375, 8.75, 17.5, and 35 mmol/L) over 24, 48, and 96 h, integrating histology, ultrastructure, gene expression (RT-qPCR), and non-specific immune enzyme assays. Histopathological and ultrastructural analyses revealed concentration- and time-dependent damage, including vacuolization, hepatic tubule disintegration, nuclear condensation, mitochondrial reduction, and loss of cellular integrity. Molecular analysis demonstrated upregulation of genes associated with the TLR2-MyD88-NF-κB pathway and inflammatory genes (*LITAF*, *IL-16*), alongside increased *HSP70* expression, confirming severe inflammation and cellular stress. Furthermore, apoptosis was induced via upregulated *Bax* and *Caspase-3*, downregulated *Bcl-2*, and DNA fragmentation. Non-specific immune responses in the hepatopancreas exhibited dynamic changes: acid phosphatase (ACP) was initially activated at low alkalinity but inhibited at high concentrations, while alkaline phosphatase (AKP) activity increased at 96 h. Notably, the hepatopancreas proved more sensitive to this stress than the hemolymph. Collectively, carbonate alkalinity causes multidimensional hepatopancreatic injury in *E. sinensis* through structural disruption, inflammation mediated by TLR2-MyD88-NF-κB signaling pathway-related genes, apoptosis induction, and immune enzyme dysregulation, posing a significant threat to crab health in salinized waters.

## Simple Summary

Rising salt levels in freshwater worldwide threaten aquatic animals like Chinese mitten crabs. We studied how carbonate alkalinity harms the crabs’ key immune and digestive organ, the hepatopancreas. Crabs were exposed to different alkalinity levels for up to 96 h. Higher alkalinity caused severe damage: cells swelled, developed empty spaces, and their structure broke down. Inside the cells, vital parts like energy-producing mitochondria shrank or vanished, and the nucleus condensed. This stress activated harmful inflammation pathways and increased cell death signals while reducing protective signals. We also found that an important stress-response protein (HSP70) surged. The non-specific immune system in the hepatopancreas reacted strongly—some increased initially but dropped under high stress, while others rose over time. Crucially, the hepatopancreas was more sensitive to this stress than the blood. Our work shows that carbonate alkalinity damages crabs by destroying tissue, causing inflammation, triggering cell death, and disrupting enzymes. This multifaceted harm threatens crab survival in increasingly saline waters, highlighting risks to freshwater ecosystems and aquaculture.

## 1. Introduction

Saline–alkaline water represents a complex aquatic ecosystem characterized by high alkalinity, wide salinity ranges, and intricate ionic compositions, often involving unbalanced ion ratios and seasonal fluctuations [1]. China’s low-lying saline–alkaline water areas cover 4.6 × 10^5^ square kilometers, which are widely distributed in northeast China, northwest China, and most northern regions [2,3]. The high pH, complex ionic composition, and ionic imbalance in these waters significantly disrupt the physiological homeostasis of aquatic animals, inhibiting growth and survival while reducing production efficiency [4,5,6,7]. Among the various stressors, carbonate alkalinity (primarily CO_3_^2−^ and HCO_3_^−^) is a critical factor threatening aquatic animal survival. These ions can enter organisms via the gills and intestine, disrupting osmoregulation and internal homeostasis [8,9,10]. Accumulating evidence demonstrates that excessive carbonate alkalinity causes severe damage to various crustacean and fish species. For instance, it impairs gill tissue damage, ion transport dysfunction, and cellular apoptosis in *Litopenaeus vannamei* [11]; disrupts the hepatopancreatic lumen and vacuolar structures in *Macrobrachium nipponense* [12]; and impairs the antioxidant system in the hepatopancreas of *E. sinensis* [13]. Furthermore, it impairs the antioxidant system of crucian carp, triggering immune responses, gill tissue damage, and apoptosis [14]; inhibits antioxidant capacity and ammonia metabolism in Songpu mirror carp, inducing immune responses [2]; and leads to reactive oxygen species (ROS) accumulation in Nile tilapia, compromising the antioxidant system [7]. Although some studies (e.g., Song et al. [15]) report that Nile tilapia exhibits good adaptability and growth performance in specific saline–alkaline water concentrations, the majority of evidence indicates that carbonate alkalinity poses a serious stress risk to aquatic organisms.

The TLR2-MyD88-NF-κB pathway is a key signaling cascade mediating inflammatory responses. Upon exposure to external stimuli such as pathogen-associated molecular patterns (PAMPs) or damage-associated molecular patterns (DAMPs), this pathway is activated in specific tissues or cells. Toll-like receptor 2 (TLR2), functioning as a pattern recognition receptor (PRR), recognizes these molecular patterns and initiates downstream signaling [16,17]. The signal is transduced via the adaptor protein MyD88 to the core transcription factor NF-κB, promoting its nuclear translocation and subsequent transcription of pro-inflammatory cytokine genes [18,19,20]. Notably, dysregulated TLR2 activation is implicated not only in the initiation and progression of inflammation but also in specific pathological processes, such as liver injury [21,22]. For instance, our previous study demonstrated that soybean agglutinin induces hepatic inflammation in Ussuri catfish by activating the TLR2-MyD88-NF-κB pathway [18]. Similarly, Feng et al. reported that Cu^2+^ exposure triggers hepatopancreatic inflammation in Chinese mitten crabs through the same mechanism [22]. Consequently, the activation level of this pathway serves as a reliable indicator of liver inflammatory response severity.

The Chinese mitten crab (*Eriocheir sinensis*) is an economically significant crustacean species in China, widely distributed throughout Southeast Asia. It is prized for its palatable meat, high nutritional value, and unique flavor [23,24]. The hepatopancreas serves as both the primary edible portion and the central immune organ in *E. sinensis*, playing a dual critical role in its physiology: it facilitates the synthesis and storage of nutrients while also functioning as the core site for producing and metabolizing innate immune molecules. This organ performs high-intensity antioxidant defense and diverse metabolic reactions [25,26]. Notably, *E. sinensis* exhibits a benthic lifestyle, making it highly susceptible to stressors from aquatic environmental factors—such as water quality deterioration and pollutant accumulation—which compromise its health and reduce aquaculture productivity [27]. Although numerous successful cases exist for cultivating fish and shrimp species in saline–alkaline waters [28], research on the adaptability and physiological responses of *E. sinensis* to carbonate alkalinity stress remains comparatively limited.

Therefore, this study evaluated hepatopancreatic damage in *E. sinensis* induced by carbonate alkalinity stress, determined inflammatory responses via TLR2-MyD88-NF-κB-related genes, and assessed apoptosis and immune enzyme responses to elucidate the underlying mechanisms. This research provides important theoretical insights and practical applications for promoting sustainable aquaculture development.

## 2. Materials and Methods

### 2.1. Ethics Statement

All experiments were conducted under the guidance of the Care and Use of Laboratory Animals in China. This research was approved by the Committee on the Ethics of Animal Experiments of Northeast Agricultural University (Code: NEAUEC20230262, 413 Date: 12 March 2026).

### 2.2. Experimental Animals

Experimental *E. sinensis* specimens were obtained from a commercial farm in Panjin, Liaoning Province, China. Prior to experimentation, crabs underwent a 15-day acclimation period in holding tanks and were fed a commercial diet. Environmental conditions were maintained as follows: water temperature at 26.0 ± 1.0 °C, dissolved oxygen (DO) > 5 mg/L, and a 12 h light–12 h dark photoperiod provided by fluorescent lighting. Throughout the acclimation period, one-third of the tank water was replaced daily with aerated tap water. Crab health was monitored rigorously, and any mortalities were promptly removed.

### 2.3. Experimental Design

The experimental design consisted of a control group and four alkalinity treatment groups. The control group utilized fully aerated tap water. Alkalinity gradients for the treatment groups (4.375, 8.75, 17.5, and 35 mmol/L NaHCO_3_) were established based on the 96 h LC_50_ value for *E. sinensis* under alkaline conditions, determined in preliminary research by our group [8]. These concentrations corresponded to 1/16, 1/8, 1/4, and 1/2 of the LC_50_ value, respectively. Treatment water was prepared by adding analytical-grade sodium bicarbonate to aerated tap water, with alkalinity verified using hydrochloric acid titration with methyl orange indicator.

Each alkalinity level was tested in triplicate, with 30 *E. sinensis* per replicate. The body weight was 17.5 ± 0.5 g, with a female-to-male ratio of 1:1. To maintain stable water quality, feeding was suspended during experiments, and 100% water exchange was performed daily. Water quality was monitored daily throughout the experiment, with average parameters recorded as follows: temperature (26 ± 1 °C), dissolved oxygen (>5 mg L^−1^), pH (7.6–8.2), NH_3_-N (<0.1 mg L^−1^), and NO_2_^−^-N (<0.1 mg L^−1^).

### 2.4. Sample Collection

At 24, 48, and 96 h post-experiment initiation, ten *E. sinensis* individuals were randomly sampled per tank. Sampling procedures included (1) hemolymph extraction, followed by (2) rapid dissection to isolate hepatopancreas tissue. The excised hepatopancreas was divided into two portions. One portion was immediately flash-frozen in liquid nitrogen and stored at −80 °C for subsequent gene expression analysis. The other portion was aliquoted and stored at −80 °C for later enzyme activity assays. In addition, for the experimental crabs subjected to 96 h alkali stress, small pieces of hepatopancreas tissue, approximately 1 mm^3^, were quickly cut with a scalpel and immediately placed in 2.5% glutaraldehyde fixative for transmission electron microscopy (TEM) observation; meanwhile, another part of the hepatopancreas tissue was placed in 10% formalin fixative for subsequent paraffin embedding and tissue section preparation. The preparation and observation of hematoxylin–eosin (HE) staining [29] and TEM (HT7800, Hitachi, Tokyo, Japan) [13,30] were carried out with reference to existing methods. The organ index (I_org_) was applied to evaluate hepatopancreatic injury in *E. sinensis* under carbonate alkalinity stress. The calculation of I_org_ was performed according to the method described by de Melo et al. [31].

### 2.5. RT-qPCR Analysis of Hepatopancreas Tissue

Total RNA was extracted from hepatopancreas tissue of each replicate group using the RNAiso Plus Kit (Takara, Catalog No.: 9109) (Beijing, China) according to the manufacturer’s instructions. RNA quality and concentration were assessed by measuring absorbance at 260/280 nm (OD 260/280). After detection, samples with OD values between 1.8 and 2.0 were selected, and cDNA was synthesized using the PrimeScript RT Reagent Kit with gDNA Eraser (Takara, Catalog No.: RR047A) (Beijing, China), which was stored at −20 °C for real-time fluorescent quantitative PCR (RT-qPCR) detection.

RT-qPCR was performed using the TB Green^®^ Premix Ex Taq™ II Kit (Takara, Catalog No: RR820A) (Beijing, China). The total reaction volume was 20 μL, containing 10 μL of TB Green Premix Ex Taq II, 1.6 μL of forward and reverse primers, 0.4 μL of ROX Reference Dye (50×), 6 μL of RNase-free dH_2_O, and 2 μL of cDNA template. The amplification program was set as follows: the first stage at 95 °C for 30 s; the second stage with 40 cycles of 95 °C for 5 s and 60 °C for 34 s; and the third stage at 95 °C for 15 s, 60 °C for 1 min, and 95 °C for 15 s. S27 and β-actin were selected as internal reference genes, and the relative expression of the data was calculated using the 2^−ΔΔCT^ method according to our previous research [32]. The primer sequences of the genes used in the experiment are shown in Table 1.

### 2.6. DNA Ladder Detection

To assess the effect of alkali stress on hepatopancreas cell apoptosis in *E. sinensis*, total DNA was extracted from stressed hepatopancreas samples using a DNA purification kit (Beyotime, C0008) (Shanghai, China) according to the manufacturer’s protocol. DNA samples and Trans2K DNA Marker (TransGen Biotech, BM101-02) (Beijing, China) were loaded onto a 1% agarose gel. Electrophoresis was conducted in 1× TAE buffer at 150 V for 30 min. DNA bands were subsequently visualized and documented using an ultraviolet gel imaging system.

### 2.7. Determination of Non-Specific Immune Capacity

Approximately 0.5 g of hepatopancreas tissue was weighed per crab and homogenized in ice-cold physiological saline at a 1:9 (*w*/*v*) ratio. The homogenate was centrifuged at 8000× *g* for 15 min at 4 °C. The resulting supernatant (hepatopancreas homogenate supernatant) was collected and stored at −20 °C for subsequent analysis. After hemolymph collection, the samples were refrigerated for 12 h until they formed a jelly-like state. The hemolymph was fully homogenized with a needle and then centrifuged at 8000 rpm and 4 °C for 15 min. The supernatant hemolymph was collected and stored at −80 °C for subsequent assays.

According to the kit instructions from Nanjing Jiancheng Bioengineering Institute (Nanjing, China), the activities of acid phosphatase (ACP) and alkaline phosphatase (AKP) in the supernatant of hepatopancreas homogenate and plasma were, respectively, determined by spectrophotometry (colorimetric method). The specific operating steps and reaction system were carried out in accordance with the kit instructions. After the reaction was completed, a spectrophotometer was used to measure the absorbance value and calculate the enzyme activity unit.

### 2.8. Statistical Analysis

All data were analyzed for differences between groups using one-way analysis of variance (ANOVA) and Duncan’s multiple-comparison test. The analysis was performed using the SPSS 25 program (SPSS Inc., Chicago, IL, USA), suitable for the Windows operating system. A *p*-value < 0.05 indicated a significant difference, and the data results are expressed as mean ± SEM.

## 3. Results

### 3.1. Carbonate Alkalinity Stress Causes Damage to the Hepatopancreas Tissue Structure

**Figure 1:**
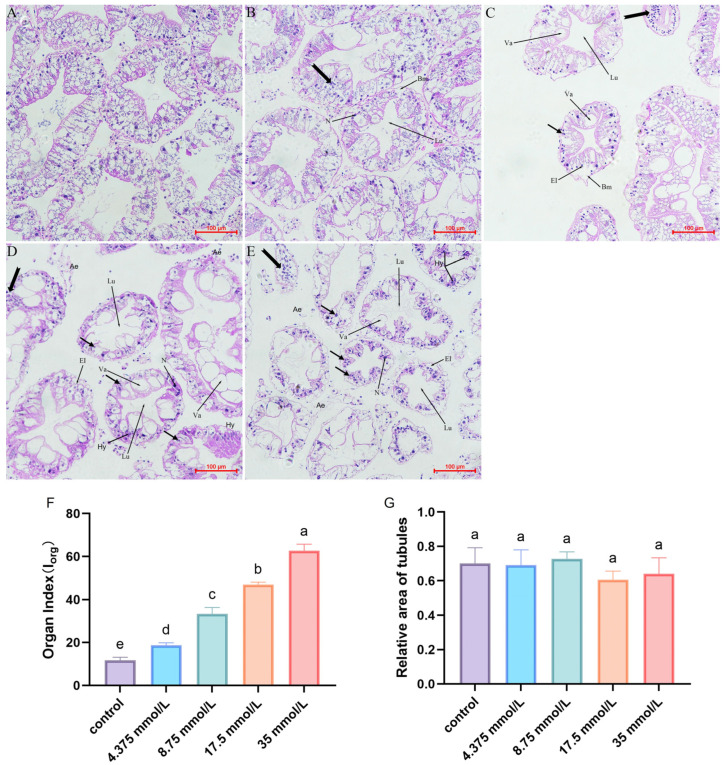
Histopathological changes in the hepatopancreas of *Eriocheir sinensis* exposed to carbonate alkalinity (HE staining, *n* = 3): (**A**) control group; (**B**) 4.375 mmol/L group; (**C**) 8.75 mmol/L group; (**D**) 17.5 mmol/L group; (**E**) 35 mmol/L group. (**F**): Mean ± SEM of organ index; (**G**) relative area of tubules. Lu: tubular lumen; Bm: basement membrane; Va: vacuole; EI: epithelial cells; N: nucleus; Hy: hyperplasia—aggregation of hematocytes (double arrowhead); Ae: atrophied epithelium—aggregation of hematocytes (double arrowhead), evidence of necrotic processes (arrow). Different lowercase letters indicate significant differences among groups (*p* < 0.05). The relative area of the tubules is calculated as the tubular lumen area divided by the total area.

- In the control group (Figure 1A), the hepatopancreatic tubules were intact with closely arranged epithelial cells.
- In the 4.375 mmol/L alkali group (Figure 1B), the tissue structure remained largely normal, although some epithelial cells had detached from the basement membrane.
- With increasing alkali concentration, tissue damage became more pronounced. In the 8.75 mmol/L group (Figure 1C), cellular vacuolization was observed, accompanied by the disintegration of some epithelial cells and deformation of the tubular lumen. However, there was no significant difference in the relative area of the hepatopancreatic tubules compared to the control group (*p* > 0.05, Figure 1G).
- In the 17.5 mmol/L group (Figure 1D), the tubular lumen was enlarged and deformed, the number of vacuoles increased, and nuclear aggregation and pyknosis became evident; however, the relative area of the hepatopancreatic tubules was not significantly different from that of the control group (*p* > 0.05, Figure 1G).
- Finally, in the 35 mmol/L group (Figure 1E), severe structural destruction was observed, characterized by extensive epithelial cell disintegration, nuclear aggregation and pyknosis, and a severely distorted lumen.
- With the increase in carbonate alkalinity, the organ index in all stress groups showed significant differences compared to the control group (*p* < 0.05, Figure 1F). The highest value was observed in the 35 mmol/L group, indicating that hepatopancreatic damage increased in a concentration-dependent manner.

### 3.2. Carbonate Alkalinity Stress Causes Ultrastructural Damage to the Hepatopancreas

**Figure 2:**
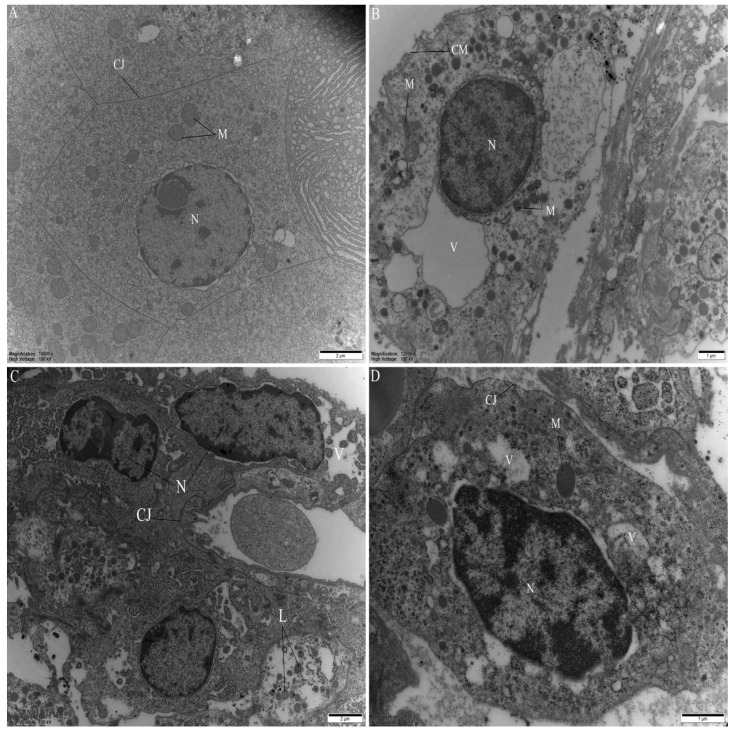
Transmission electron microscopy results of the hepatopancreas of *Eriocheir sinensis*: (**A**) control group; (**B**) 8.75 mmol/L group; (**C**,**D**) 35 mmol/L group. CJ: cell junctions; M: mitochondria; N: nucleus; V: vacuole; CM: cell membrane; L: lysosome.

- The hepatopancreas cells in the control group (Figure 2A) have intact structures, with normal morphology in mitochondria, neatly arranged microvilli, intact and clear cell boundaries, and tight connections between cells.
- Under the alkalinity condition of 8.75 mmol/L for 96 h (Figure 2B), some mitochondria change in shape and become invaginated, the morphology of cell boundaries changes, the nuclear substances begin to condense towards the nuclear membrane, and vacuoles appear in the cells.
- Under the alkalinity conditions of 35 mmol/L for 96 h (Figure 2C,D), the chromatin in the nucleus condenses and distributes along the nuclear membrane, the morphology of the nucleus undergoes severe changes, the microvilli protrusions of the cells disappear, the cell connections are not obvious, and typical characteristics of cell apoptosis appear.

### 3.3. Carbonate Alkaline Stress Leads to Inflammation in the Hepatopancreas

**Figure 3:**
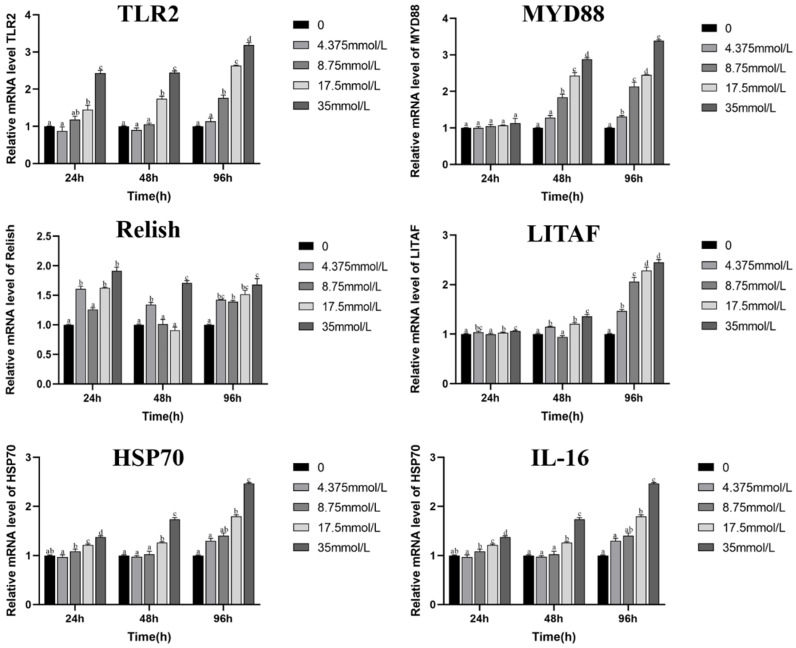
Results of the TLR2-MyD88-NF-κB pathway and other gene expression in the hepatopancreas of *Eriocheir sinensis*. Data are expressed as mean ± standard deviation (*n* = 3), and the different lowercase letters indicate significant differences among groups (*p* < 0.05).

As shown in Figure 3, under carbonate alkalinity stress, the expression levels of *TLR2*, *MyD88*, *IL-16*, and *HSP70* in the hepatopancreas of *E. sinensis* exhibited an upward trend with increasing time and alkalinity. Notably, the expression levels in the 35 mmol/L group at 96 h were significantly higher than those in the control group (*p* < 0.05). In contrast, the expression of *Relish* initially decreased and then increased across the concentration and time gradients, peaking in the 35 mmol/L group at 96 h. Regarding *LITAF*, its expression initially decreased and then increased with rising alkalinity at 24 h and 48 h; however, at 96 h, the expression level increased consistently with alkalinity, reaching a value significantly higher than that of the control group (*p* < 0.05).

### 3.4. Carbonate Alkalinity Stress Leads to Apoptosis in the Hepatopancreas

**Figure 4:**
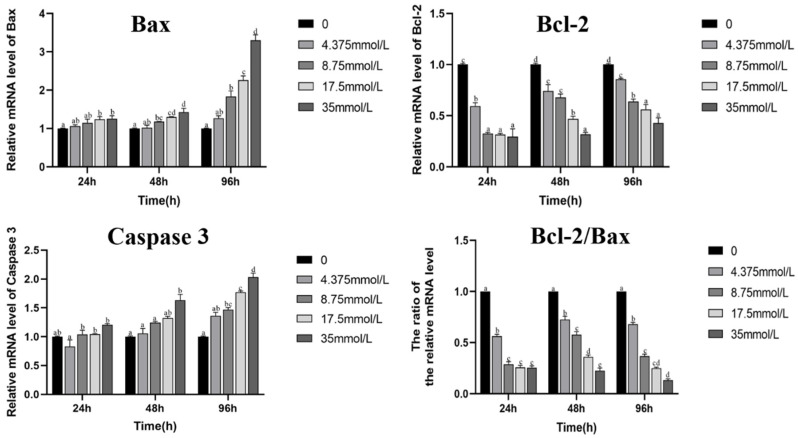
Effect of carbonate alkaline stress on the expression of apoptotic genes in the hepatopancreas of *Eriocheir sinensis*. Data are expressed as mean ± standard deviation (*n* = 3), and the different lowercase letters indicate significant differences among groups (*p* < 0.05).

**Figure 5:**
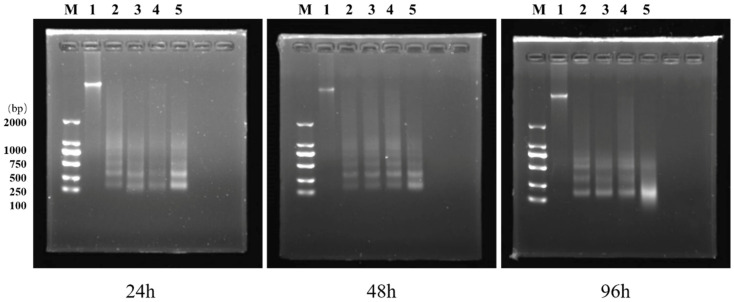
The DNA ladder method was used to detect the occurrence of apoptosis induced by different alkalinity levels in the hepatopancreatic cells of *Eriocheir sinensis*. M: marker of 2000 bp length; 1: control group; 2: 4.375 mmol/L group; 3: 8.75 mmol/L group; 4: 17.5 mmol/L group; 5: 35 mmol/L group (*n* = 3).

- As shown in Figure 4, the expression levels of *Bax* and *Caspase-3* in the hepatopancreas showed an upward trend with the increase in alkalinity and the extension of treatment time: they were slightly higher than those in the control group at 24 h and 48 h and significantly increased at 96 h (35 mmol/L group, *p* < 0.05).
- The expression level of *Bcl-2* showed a downward trend (Figure 4).
- The results of DNA ladder detection (Figure 5) showed that DNA fragmentation gradients appeared in the hepatopancreas of each treatment group, confirming that carbonate alkaline stress induced cell apoptosis, and the degree of fragmentation intensified with the increase in alkalinity.

### 3.5. Effects of Carbonate Alkalinity Stress on the Activity of Nonspecific Immune Enzymes

**Figure 6:**
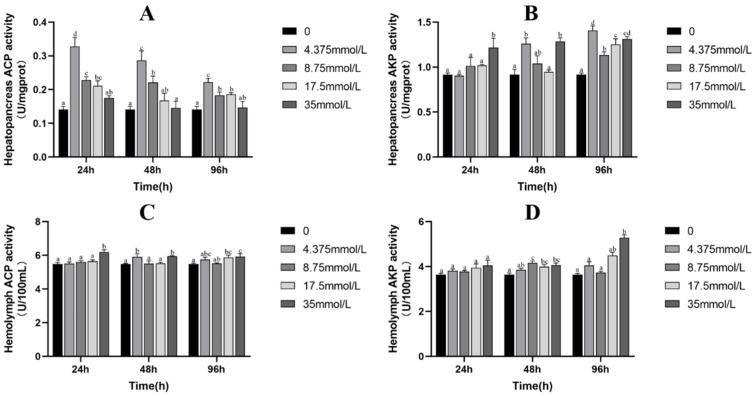
Results of non-specific immunoenzyme activity assay in *Eriocheir sinensis*: (**A**) hepatopancreas acid phosphatase (ACP) activity; (**B**) hepatopancreas alkaline phosphatase (AKP) activity; (**C**) hemolymph ACP activity; (**D**) hemolymph AKP activity. Data are expressed as mean ± standard deviation (*n* = 3), and the different lowercase letters indicate significant differences among groups (*p* < 0.05).

- The ACP activity of the hepatopancreas increased significantly after 24 h and 48 h of carbonate-alkali stress, but there was no significant difference between the 35 mmol/L group and the control group (*p* > 0.05).
- The ACP activity of the hepatopancreas decreased slightly after 96 h of alkali stress but was still significantly higher than that of the control group, except for the 35 mmol/L group (*p* < 0.05).
- It is worth noting that the ACP activity in the hemolymph of the 35 mmol/L group was always significantly higher than that of the control group (*p* < 0.05) and was at the highest value.
- After 24 h of carbonate alkalinity stress, AKP activity in the hepatopancreas and hemolymph showed no significant changes, except that activity in the hepatopancreas of the 35 mmol/L group was significantly higher than the control (*p* < 0.05).
- In the hepatopancreas at 48 h and 96 h, activity in the 4.375 mmol/L group was significantly higher than in the control. Subsequently, activity exhibited an initial decrease followed by an increase with rising alkalinity. At 48 h, activity in the 8.75 and 17.5 mmol/L groups showed no significant difference from the control (*p* > 0.05) but was significantly higher in all other groups (*p* < 0.05).
- In the hemolymph at 48 h, activity was significantly higher than the control in all groups except the 4.375 mmol/L group (*p* < 0.05). At 96 h, activity in the 4.375, 8.75, and 17.5 mmol/L groups showed no significant difference from the control (*p* > 0.05), while it was significantly higher in the 35 mmol/L group (*p* < 0.05) (Figure 6).

## 4. Discussion

Global soil salinization severely restricts agricultural development [33] and exacerbates the depletion of freshwater resources [15]. In recent years, the development and utilization of saline–alkaline water have gradually become a new challenge faced by the global aquaculture industry. Researchers have carried out preliminary explorations on saline–alkaline water aquaculture. At present, there are successful cases of culturing *Litopenaeus vannamei* [34], *Oryzias latipes* [35], and *Acipenser baerii* [36] in saline–alkaline waters, but there are few reports on the culture of *E. sinensis* in saline–alkaline water. Carbonate alkalinity stress is the core type and main manifestation of alkali stress. Based on our multi-dimensional evaluation of hepatopancreatic damage, we identified 17.5 mmol/L as the critical concentration for acute toxicity and 8.75 mmol/L as the safe concentration. However, these thresholds serve only as preliminary indicators. Additional research is therefore necessary to validate these limits for long-term aquaculture applications.

Previous studies have demonstrated that elevated carbonate alkalinity induces severe cellular damage in the gill tissues of aquatic animals. For example, Gao et al. reported that excessively high HCO_3_^−^ in water led to the fusion and detachment of gill epithelial cells in *Leuciscus waleckii* [37]. Another example is that Wang et al. reported that acute carbonate alkaline stress caused severe deformation of gill lamellae, hyperplasia of the outer epithelium, and edema in *Oreochromis niloticus* [38]. Furthermore, Yu et al. also found that carbonate alkalinity inhibits the growth performance and hepatic antioxidant capacity of Nile tilapia while increasing the proportion of pathogenic bacteria in the intestine [39]. It is worth noting that there are still research gaps in the mechanism of carbonate alkalinity-induced hepatopancreas damage in crustaceans. The hepatopancreas is the main organ for metabolism, detoxification, and immunity in crustaceans [40,41], and its health is crucial to the survival of crustaceans. This study found that after the carbonate alkalinity reaches 17.5 mmol/L, the damage to the hepatopancreas cells of *E. sinensis* gradually intensifies. When the alkalinity reaches 35 mmol/L, a large number of nuclear aggregations and obvious cell vacuolation even appear. These results confirm that high-concentration carbonate alkalinity severely disrupts hepatopancreatic tissue architecture in *E. sinensis*, thereby addressing a critical gap in our understanding of alkali stress toxicity in crustaceans.

The inflammatory response is a physiological reaction of organisms to exogenous stimuli and pathogens, and it is a non-specific process involving immune cells to repair the body [42]. TLR2-MyD88-NF-κB is a classic pro-inflammatory signaling pathway and a core regulatory hub for the body’s response to exogenous stimuli to induce inflammation. NF-κB plays a crucial role in regulating the transcription of inflammatory factors [43]. In crustaceans, the NF-κB pathway similarly regulates cytokine production [44,45]. Relish, an NF-κB-like transcription factor predominantly expressed in the hepatopancreas, mediates immune and inflammatory responses to external stressors [46]. TLR and LITAF are important components of the NF-κB pathway, which can regulate the production of inflammatory factors and induce the occurrence of inflammatory responses [47]. Our study found that with the increase in carbonate alkalinity and stress time, the expression levels of genes related to the TLR2-MyD88-NF-κB signaling pathway also increased, accompanied by the elevation of inflammatory cytokines *Il16* and *LITAF*. This indicates that this signaling pathway is relatively sensitive to carbonate alkalinity. It also suggests that carbonate alkalinity stress induces inflammation by upregulating the genes related to the TLR2-MyD88-NF-κB signaling pathway. It is worth noting that at the 48 h stress node, the gene expression levels of *TLR2*, *Relish*, *LITAF*, and *IL-16* in the 8.75 mmol/L group showed no significant difference compared with the control group, indicating that the carbonate alkalinity of 8.75 mmol/L is relatively safe.

Apoptosis is an active form of programmed cell death characterized by caspase cascade activation, cellular shrinkage, chromatin condensation, and DNA fragmentation [48]. As a core player in this process, caspase-3 (a cysteine–aspartate protease) drives apoptosis by cleaving key intracellular substrates [49]. Bax and Bcl2 both belong to the Bcl2 protein family. They antagonistically regulate the initiation of the mitochondrial apoptotic pathway, among which Bax is a pro-apoptotic protein [50], and Bcl2 is an anti-apoptotic protein [51]. According to researchers, drastic changes in environmental factors in the aquatic environment where aquatic animals live can cause cell apoptosis, such as hypoxic environments [52] and excessively high levels of ammonia nitrogen in water [53]. Similarly, we found that exposure to an aquatic environment with high carbonate alkalinity also induces apoptosis. In our previous study, it was confirmed that exposure to high doses of carbonate alkalinity can induce apoptosis in hepatopancreas cells of *E. sinensis* through the ROS/MAPK signaling pathway, which is manifested by a significant increase in the expression levels of *Bax*/*Bcl2* and *caspase-3* [54]. The present study not only corroborates these findings but also provides direct ultrastructural evidence of pronounced apoptotic morphology in the high alkalinity group. Furthermore, DNA ladder assays revealed severe DNA fragmentation in hepatopancreatic tissues following a 96 h exposure to 35 mmol/L carbonate alkalinity. Collectively, these observations offer robust molecular and structural confirmation of carbonate alkalinity-induced hepatopancreatic apoptosis in this species.

The non-specific immune system serves as the primary defense against pathogen invasion, and its homeostasis is critical for maintaining the health of aquatic organisms [55]. Among them, AKP and ACP are key biomarkers reflecting the immune function of aquatic animals [56]. In this study, carbonate alkalinity stress induced significant alterations in the activities of these enzymes within both the hepatopancreas and hemolymph. This observation aligns with previous reports indicating that drastic environmental fluctuations can disrupt non-specific immunity, thereby predisposing aquatic animals to various health complications [56,57]. Further observation revealed that the changes in the activities of AKP and ACP enzymes in hemolymph were not as significant as those in the hepatopancreas, which may reflect that the hepatopancreas is more sensitive to carbonate alkaline stress. This is consistent with extensive evidence identifying the hepatopancreas as the principal site of ACP and AKP activity in crustaceans [58]. Indeed, histochemical studies have successfully localized these enzymatic activities within hepatopancreatic tissues, providing direct structural evidence for their essential physiological roles in this organ [59,60], which provides direct histological evidence for their important physiological roles in this organ. It is worth noting that the activity of the ACP and AKP enzymes in the hepatopancreas showed different changing trends. We speculate that this is due to the difference in the optimal pH of AKP and ACP enzymes; that is, affected by carbonate alkaline stress, the pH of the microenvironment in the body of *E. sinensis* has changed.

## 5. Conclusions

Firstly, this study successfully constructed a model of hepatopancreas injury in *E. sinensis* induced by carbonate alkalinity. Through histopathological and ultrastructural analyses, it was clarified that carbonate alkalinity concentrations above 17.5 mmol/L were associated with marked hepatopancreatic alterations, inflammatory responses, apoptosis-related gene expression, and immune enzyme disturbances. Through DNA ladder detection and observation of apoptotic ultrastructures, direct molecular and morphological evidence was provided for the induction of hepatopancreas cell apoptosis by high alkali stress (35 mmol/L). However, this study also has certain limitations, such as the lack of investigation into long-term carbonate alkalinity stress and the absence of water conductivity measurements. In summary, this study evaluated the potential risk threshold of acute carbonate alkalinity by assessing indicators such as inflammation pathway activation, cellular apoptosis, and immune enzyme dysregulation. This threshold provides a theoretical and scientific basis for safe alkalinity regulation, offering practical guidance for future commercial aquaculture.

## Figures and Tables

**Table 1:** RT-qPCR primer sequences.

Primer Names	Sequence (5′-3′)
Bax F	GTCAGTGAACCTCAGCTGCAT
Bax R	CACAGCCACATCACCCACGAA
Bcl-2 F	ATAAGGTGGTTCGCTCCGTC
Bcl-2 R	TTAACACAGTCCGAGGCCAG
Caspase 3 F	CATTCGCCAGCCTTGCCCACA
Caspase 3 R	TCTGTCGTGGTTCCTTGTAGCCTT
Hsp70 F	GGCAAGGCAGCGAAGGTCATC
Hsp70 R	CGGCATTGGTGACAGACTGACG
LITAF F	TAAAGGCAAGGGAGGCTTCG
LITAF R	GAATGGAGCTTGAGGTGGCA
Relish F	TCTCCCTACTCTGACCATTCC
Relish R	TTCCCACCATCTCACTCTTGT
MyD88 F	GCCATCGCAGTCGCCAAGTT
MyD88 R	GGCATCCTGTTCATCCAGTTCTGAC
IL-16 F	TGAACGAAGGGAGGTACAAC
IL-16 R	ATGCCCTGAGAGTAGCTTGA
TLR2 F	CATACCAGGACGACGAAC
TLR2 R	AGACATTGAGCGAGGAGA
S27 F	CCCCCAAGAAGATCAAGCACA
S27 R	CAGATGGCAGCGACCACAGT
β-actin F	TCGTGCGAGACATCAAGGAAA
β-actin R	AGGAAGGAAGGCTGGAAGAGTG

**Note:** F, Forward; R, Reverse.

## Data Availability

The raw data supporting the conclusions of this article will be made available by the authors upon request.

## Abbreviations

The following abbreviations are used in this manuscript:

ACP	Acid phosphatase
AKP	Alkaline phosphatase
TLR2	Toll-like receptor 2
*E. sinensis*	*Eriocheir sinensis*
TEM	Transmission electron microscopy
RT-qPCR	Real-time fluorescent quantitative PCR
